# Potential Antioxidative and Anti-Hyperuricemic Components Targeting Superoxide Dismutase and Xanthine Oxidase Explored from *Polygonatum Sibiricum* Red.

**DOI:** 10.3390/antiox11091651

**Published:** 2022-08-25

**Authors:** Jing Li, Zhi Wang, Minxia Fan, Guangwan Hu, Mingquan Guo

**Affiliations:** 1 Key Laboratory of Plant Germplasm Enhancement and Specialty Agriculture, Wuhan Botanical Garden, Chinese Academy of Sciences, Wuhan 430074, China; 2 College of Life Sciences, University of Chinese Academy of Sciences, Beijing 100049, China; 3 College of Pharmacy, Hunan University of Chinese Medicine, Changsha 410208, China; 4 Sino-Africa Joint Research Center, Chinese Academy of Sciences, Wuhan 430074, China; 5 Innovation Academy for Drug Discovery and Development, Chinese Academy of Sciences, Shanghai 201203, China

**Keywords:** *Polygonatum sibiricum*, UF-LC-MS, antioxidative, anti-hyperuricemic, SOD, XOD

## Abstract

*Polygonatum sibiricum* Red. (*P. sibiricum*) has been used as a traditional Chinese medicine with a wide range of pharmacology effects. However, the responsible bioactive compounds and their mechanisms of action concerning its antioxidative and anti-hyperuricemic activities remain unexplored. In this work, the antioxidant capacity of *P. sibiricum* was firstly evaluated with the 2,2-diphenyl-1-picrylhydrazyl (DPPH), 2,2’-azinobis-(3ethylbenzthiazoline)-6-sulfonic acid (ABTS) and ferric-reducing antioxidant power (FRAP) assays, from which the ethyl acetate (EA) fraction exhibited the highest DPPH, ABTS radical scavenging, and ferric-reducing capacities. Meanwhile, the EA fraction displayed the highest total phenolic and flavonoid contents among the four fractions. Next, the potential ligands from the EA fraction were screened out by bio-affinity ultrafiltration liquid chromatography-mass spectrometry (UF-LC-MS) with superoxide dismutase (SOD) and xanthine oxidase (XOD). As a result, *N*-*trans*-*p*-coumaroyloctopamine, *N*-*trans*-feruloyloctopamine, *N*-*trans*-feruloyltyramine were identified as potential SOD ligands, while *N*-*cis*-*p*-coumaroyltyramine was determined as potential XOD ligand. Additionally, these four ligands effectively interact with SOD and XOD in the molecular docking analysis, with binding energies (BEs) ranging from –6.83 to –6.51 kcal/mol, and the inhibition constants (Ki) from 9.83 to 16.83 μM, which were better than the positive controls. In conclusion, our results indicated that *P. sibiricum* has good antioxidative and anti-hyperuricemic activities, and its corresponding active ligands targeting SOD and XOD could be explored by the UF-LC-MS method.

## 1. Introduction

*Polygonatum sibiricum* Red. belonging to the Liliaceae family has been distributed in the temperate northern hemisphere countries, such as China, Japan, Korea, India, Russia, Europe, and North America [1,2]. In China, *P. sibiricum* is well-known as a traditional medicinal herb and functional food [3,4], as well as a health-improving substance [5], with a sweet fragrance and taste. *P. sibiricum* has been included in the “list of items that are both food and medicine” by the National Health Commission of the People’s Republic of China. It was initially described as replenishing Qi and nourishing Yin, strengthening the spleen, and nourishing the lungs and kidneys in “Special Records of Famous Doctors”. Thus far, it has been reported to have a variety of pharmacological applications and biological activities, including antioxidative and anti-hyperuricemic activities. Scientific reports have supported its anti-osteoporosis [6], neuroprotective [7], immunity enhancement [8], anti-diabetic [9], anti-aging [10], anti-cancer [11], and other effects. The principal mechanisms of these accounted biological activities have been attributed to multiple natural phytochemicals, such as alkaloids [12], flavones [13], steroid saponins [14], lignans [15], amino acids [16], and polysaccharides [17]. Due to the species’ richness in various chemical components and its function as a medicinal and food plant resource, *P. sibiricum* has been extensively investigated in recent years.

Reactive oxygen species (ROS) are cellular metabolic products, categorized as free radicals (superoxide anion radicals (O_2_^•−^) and non-free radical species (hydrogen peroxide (H_2_O_2_) [18], and they are necessary for many important life processes, such as cellular growth, proliferation and differentiation, energy supply, and the aging of some organisms [19]. Oxidative stress is caused by the imbalanced amounts of ROS, which damages many organs in the human body [20], while superoxide dismutase (SOD) and xanthine oxidase (XOD) enzymes play a vital role in regulating the ROS equilibrium. Superoxide dismutase (SOD) has been recognized as a major component of the defense system against oxidative stress generated by ROS [21]. In the presence of transition metal ions, it can catalyze the conversion of O_2_^•−^ to H_2_O_2_ and oxygen (O_2_), and H_2_O_2_ can be decomposed into a hydroxy radical (·OH) and hydroxide ion (OH^−^) [22]. Hence, SOD is critical for the maintenance of the balance between oxidation and anti-oxidation. Xanthine oxidase (XOD) is a crucial flavoprotein enzyme, which participates in the catalysis of xanthine to produce uric acid and O_2_^•−^ [23]. Moreover, the O_2_^•−^ generated by excessive XOD activity is related to hyperuricemias, gouts, hepatitis, carcinogenesis, aging [18], cardiovascular diseases [24], mental disorders [25], and chronic obstructive pulmonary diseases [26]. Therefore, SOD and XOD are promising targets for the treatment of diseases related to oxidative damage [27]. Several studies have illustrated that *P. sibiricum* could exhibit antioxidant activities [28,29], while the responsible compounds leading to the specific pharmacological activities, such as antioxidation and anti-hyperuricemia, as well as their screening method based on enzymes targeting have not been reported so far. At present, the bio-affinity ultrafiltration liquid chromatography-mass spectrometry (UF-LC-MS) has been actively developed and applied to simultaneously screen out and identify potential ligands towards biological targets from complex samples [30]. It is therefore promising to screen the underlying bioactive compounds from *P. sibiricum* that tackle the oxidative damage and related diseases, such as hyperuricemia, using XOD and SOD as targets.

In this context, the active fraction among the different samples [ethanol crude extract (CE), petroleum ether (PE), ethyl acetate (EA), n-butanol (n-Bu), and aqueous (WA) fractions] should be screened and selected due to the chemical complexity and diversity of *P. sibiricum*. Thus, the antioxidant capacity of *P. sibiricum* was firstly evaluated using three different assays, DPPH (2,2-diphenyl-1-picrylhydrazyl), ABTS (2,2’-azinobis-(3ethylbenz- thiazoline-6-sulfonic acid) and FRAP (ferric-reducing antioxidant power). Meanwhile, the total phenolic and flavonoid content (TPC, TFC) were determined out to reveal their correlation with the antioxidant activities. Thereafter, the potential bioactive ligands from the EA fraction were screened out by UF-LC-MS targeting SOD and XOD enzymes. Additionally, molecular docking was operated to illustrate the interactions between the active ingredients and the target enzymes. To the best of our knowledge, this is the first time the UF-LC-MS method has been applied for the bioactive compounds screening of *P. sibiricum*. More importantly, this effective approach has successfully revealed the bioactive compounds responsible for the high antioxidative and anti-hyperuricemic activities of *P. sibiricum*, which provide a valuable reference for future study on other natural products resources.

## 2. Materials and Methods

### 2.1. Plant Materials and Preparation of Samples

The rhizomes of *Polygonatum sibiricum* Red. were collected at Huaihua, Hunan Province of China, and were authenticated by Prof. Guangwan Hu, a senior taxonomist of Wuhan Botanical Garden, Chinese Academy of Sciences. The voucher specimen (No.202201-17) was preserved in the herbarium of the Key Laboratory of Plant Germplasm Enhancement and Specialty Agriculture. The dried and pulverized rhizomes of *P. sibiricum* (1500 g) were extracted in ethanol (70%, 15 L) at room temperature for three times (2 days each time), and then concentrated to obtain the crude extract (CE, 150 g). The crude extract (150 g) was suspended in H_2_O (2.5 L) and successively partitioned with petroleum ether (2.5 × 3 L), ethyl acetate (2.5 × 3 L) and n-butanol (2.5 × 3 L) to yield PE (4.9 g), EA (3.4 g), n-Bu (14.7 g), and WA (86.8 g) fractions, respectively. The obtained samples were stored in airtight vials and kept at 4 °C for further tests.

### 2.2. Chemicals and Reagents

Rutin was purchased from J&K Scientific Ltd. (Beijing, China), Folin−Ciocalteu reagent, 1,3,5-tri(2-pyridyl)-2,4,6-triazine (TPTZ), 2,2’-azinobis-(3-ethylbenzthiazoline-6-sulfonic acid) (ABTS), ascorbic acid (Vitamin C, Vc), and 6-hydroxy-2,5,7,8-tetramethylchroman-2-carboxylic acid (Trolox) were purchased from Sigma-Aldrich Corp (Shanghai, China). The acetonitrile (ACN) and methanol of HPLC grade were supplied by TEDIA Company Inc. (Fairfield, OH, USA). All other analytical solvents and chemicals were purchased from Sinopharm Chemical Reagent Co., Ltd. (Shanghai, China). Superoxide dismutase (SOD) and xanthine oxidase (XOD) were bought from Shanghai Yuanye Bio-Technology Co., Ltd. (Shanghai, China). Ultrafiltration membranes (0.5 mL, 30 kDa/10 kDa) were purchased from Millipore Co. Ltd. (Bedford, MA, USA). All aqueous solutions of ultra-pure grade for HPLC and HPLC-UV-ESI-MS/MS analyses were prepared with EPED (Nanjing EPED Technology Development Co., Ltd. Nanjing, China).

### 2.3. Evaluation of the Antioxidant Capacity of P. sibiricum

#### 2.3.1. DPPH Free Radical Scavenging Activity

The DPPH free radical scavenging activities of *P. sibiricum* samples were determined according to a previous study reported by Xu et al. [31] with slight modifications. Initially, 10 μL samples or the positive control solutions of vitamin C (46.875–3000 μM) were mixed with 190 μL of DPPH (100 μM) in a 96-well plate. The sample mixture was then incubated in the dark for 30 min at room temperature. Finally, the absorbance at 517 nm was measured with multifunctional microplate reader. Meanwhile, methanol was used as blank control in this assay, and all the samples and controls were tested in triplicate (*n* = 3). The DPPH free radical scavenging activity was determined using the formula:

DPPH-free radical scavenging effect (%) = [(A_C_ − A_S_)/A_C_] × 100 (1)

where A_C_ and A_S_ represent the absorbance value of the blank control and the tested sample or positive control, and the IC_50_ value represents the 50% inhibition ratio of the DPPH free radicals.

#### 2.3.2. ABTS Free Radical Scavenging Activity

The ABTS free radical scavenging activities of different *P. sibiricum* extracts were conducted following the method of Zhuang et al. [32] with a few minor modifications. Briefly, the working ABTS radical cation (ABTS^+^) solution was prepared by mixing equal volumes of potassium persulfate (4.9 mM in H_2_O) and ABTS (7 mM in H_2_O), which are further incubated in the dark for 12 to 16 h. The ABTS^+^ stock solution was then diluted with methanol to obtain an absorbance of 0.700 ± 0.03 at 734 nm. After that, 10 µL of appropriately diluted samples was combined with 190 μL ABTS^+^ solution, and the absorbance of the mixture solution was recorded at 734 nm after incubation in the dark for 30 min. Vitamin C and methanol were utilized as positive and blank controls, respectively. All samples and controls were tested in triplicate (*n* = 3). The results of ABTS scavenging activity were calculated as the DPPH scavenging method as described before (Equation (1)).

#### 2.3.3. Ferric-Ion-Reducing Antioxidant Power (FRAP) Assay

The FRAP assay was performed using the method of Dordevic et al. [33] with modifications. The FRAP reagent (Fe^3+^–TPTZ solution) was composed of FeCl_3_·6H_2_O (20 mM in H_2_O), TPTZ (10 mM in 40 mM HCl), and acetate buffer (300 mM, pH = 3.6) at a ratio of 1:1:10 (*v*/*v*/*v*), which was then stored at 37 °C before use. Next, 10 μL of properly diluted samples, 30 μL of distilled water, and 260 μL FRAP reagent were mixed in turn and incubated at 37°C for 10 min. The absorbance was acquired at 593 nm by triplicate tests (*n* = 3). A calibration curve was established using FeSO_4_·7H_2_O (62.5−2000 µM) with positive control (vitamin C). The activity of FRAP was expressed as mM Fe^2+^/g of the sample tested (mM Fe^2+^/g).

### 2.4. Determination of Phenolic Constituents

#### 2.4.1. Determination of Total Phenolic Content (TPC)

The TPCs of PE, EA, n-Bu, WA, and CE extracts was determined (*n* = 3) by the Folin −Ciocalteu [34] method with modifications. In short, 20 μL of the diluted sample was mixed with 20 μL Folin−Ciocalteu reagent (in pure water, 25%, *v*/*v*) and the mixture was then incubated for 3 min. After that, 100 μL of sodium carbonate (Na_2_CO_3_, 200 μM) solution was added into the reaction system and incubated in the dark at room temperature for an hour. The absorbance was recorded at 760 nm. A calibration curve was established using gallic acid as the standard. The results of TPC were expressed in milligrams of GA equivalents (GAE) per gram of the sample (mg GAE/g sample).

#### 2.4.2. Determination of Total Flavonoid Contents (TFC)

The TFCs of PE, EA, n-Bu, WA, and CE extracts were estimated (*n* = 3) using a previously described colorimetric method [35] with minor modifications. Briefly, 30 μL of appropriately diluted sample solution was mixed with 180 μL of distilled water and 20 μL of sodium nitrite solution (NaNO_2_, 5%, *w*/*v)*. After incubation for 6 min, 40 μL of aluminum chloride solution (AlCl_3_, 10%, *w*/*v*) was added and incubated for a further 6 min. Then, 60 μL of sodium hydroxide solution (NaOH, 4%, *w*/*v*) was added and reacted for 15 min, and the absorbance of the mixture was determined at 510 nm. Rutin was used as the standard, and the results were represented as milligrams of rutin equivalent (RE) per gram of dry sample (mg RE/g sample).

### 2.5. Screening of the Potential Ligands of SOD and XOD with UF-LC-MS

Potential bioactive components with high relative binding affinity to SOD and XOD were screened by the UF-LC-MS procedures, which were carried out as previous studies [36,37]. Firstly, the EA fraction (5 mg) of *P. sibiricum* was dissolved with PBS (pH = 7.4, 995 µL with 5 µL DMSO) buffer solution and ultrasonicated for 30 min, which was subjected as the tested sample solution. 80 µL of sample solution (5 mg/mL) was incubated with SOD (0.2 U/µL) or XOD (0.1 U/µL) at 37 °C in the dark for 40 min. Secondly, the incubated solutions were transferred into 30 KDa cut-off ultrafiltration membranes, centrifuged at 10,000 rpm for 10 min at 25 °C, and immediately washed three times with PBS solution (pH = 7.4, 200 µL) through centrifugation to remove non-specific binding ligands. Thirdly, 200 μL acetonitrile (90%, *v*/*v*) was added and incubated for 10 min to release the compounds binding to SOD or XOD from the enzyme-ligand complexes, followed by centrifugation at 10,000 rpm for 10 min (*n* = 3). Finally, those ultrafiltrates were dried and reconstituted with 40 µL methanol for further analysis. In addition, the inactive enzyme group, denatured in boiling water (100 °C) for 10 min, was set up as negative control, and the treatment method is consistent with the active enzyme group.

### 2.6. UPLC-Q-TOF-MS/MS Analysis

UPLC-Q-TOF-MS/MS analysis of the EA fraction from *P. sibiricum* was carried out by ultra-high-performance liquid chromatography (UPLC) coupled to quadrupole-flight mass spectrometry (Q-TOF-MS/MS, Agilent 1290 L, Agilent 6530 MS, Agilent Technologies, Santa Clara, CA, USA). The chromatographic separation was carried out on a Sunniest C18 ACQ (2.1 mm × 50 mm, 1.7 μm) column at 30 °C, with the mobile phase consisting of ultrapure water containing 0.1 percent formic acid (A) and ACN (B). The Q-TOF-MS analysis was performed in the negative-ion modes with a dual ESI source. The LC elution gradient was set as follows: 0–20 min, 5–20% B; 20–30 min, 20–30% B; 30–40 min, 30–50% B. The flow rate was then 0.2 mL/min, and the injection volume was 20 µL. The following MS parameters were set: The capillary voltage (Vcap) was 3500 V and the fragmentor voltage was 175 V, respectively. The capillary temperature was 350 °C, and the drying gas flow rate was 8 L/min. The pressure in the nebulizer was set at 35 psi. The fixed collision energies were set as 10, 20, 40 and 60 V. The Mass Hunter workstation (Agilent) with a mass range of *m/z* 100–1500 was used to obtain profile data at a rate of one spectrum per second. The compounds were identified by comparing their retention times, parent ions, and mass fragments with references and databases.

### 2.7. Molecular Docking Study

The interaction mechanism between the potential ligands and corresponding target enzymes was further explored by molecular docking using AutoDock Tools 1.5.6 and the Discovery Studio 4.1 software based on the previous approach with minor modifications [37,38,39]. Firstly, the crystallized structures of the SOD (PDB 1CBJ) and XOD (PDB 1FIQ) were downloaded from RSCB Protein Data Bank (www.rcsb.org, accessed on 31 July 2022), and the 3D structures of the ligands with the lowest energy were established by ChemBio3D Ultra 12.0. Then, the 3D structures of ligands and receptors were processed by removing the water molecules, adding the hydrogen atoms, calculating the charge, and so on by AutoDock Tools. Subsequently, the docking active sites of SOD and XOD were optimized and obtained by Discovery Studio 4.1. Thereinto, the coordinates of the active sites of SOD and XOD were (X: 6.640; Y: 23.974; Z: 58.655) and (X: 28.671; Y: 29.977; Z: 101.417), respectively. Besides, the grid box was centered on the active sites of the receptors with a dimension size of 60 Å × 60 Å × 60 Å. Finally, molecular docking analysis between ligands and receptors was performed with 50 independent runs of the genetic algorithm by AutoDock Tools with other default parameters, and then the docking conformation was ranked according to the energy score.

### 2.8. Validation of Potential Ligands Activity by UF-LC-MS

To confirm the affinity of the potential ligands towards the target enzymes, the UF-LC-MS method was employed to estimate their relative IC_50_, and its procedure was the same as described before. Trolox, recognized for its strong antioxidant activity with an IC_50_ = 2.82 mM when inhibiting SOD, was chosen as the positive control for SOD [40]. The potential ligands to SOD together with Trolox were subjected to UF-LC-MS for the determination of their corresponding BD values, therefore the relative IC_50_ of potential ligands was calculated from these relative BD values (Figure S1 and Table S1).

### 2.9. Statistical Analysis

All data in this work were expressed as mean ± standard deviation (SD) of triplicate measurements. The IC_50_ values were calculated by plotting the percentages of scavenging activities or inhibition rates against the sample concentrations (six different concentration gradients in triplicate). Different software applications were used for statistical analysis including SPSS 25.0 (IBM Corp., New York, NY, USA), Origin 2021 (OriginLab Corporation, Northampton, MA, USA), and GraphPad Prism 5.0 (GraphPad Software Inc., San Diego, CA, USA).

## 3. Results and Discussion

### 3.1. Antioxidant Activities of P. sibiricum

**Figure 1:**
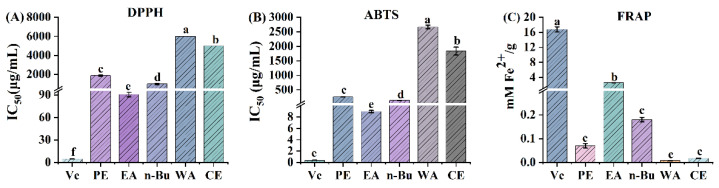
Antioxidant activity of petroleum ether (PE), ethyl acetate (EA), n-butanol (n-Bu), aqueous (WA) and crude extracts (CE) of *P. sibiricum*. (**A**) the IC_50_ value of 2,2-diphenyl-1-picrylhydrazyl (DPPH) radical scavenging assay, (**B**) the IC_50_ value of 2,2’-azinobis-(3ethylbenzthiazoline)-6-sulfonic acid (ABTS) radical scavenging assay, (**C**) ferric-ion-reducing antioxidant power (FRAP) assay. Mean values with different letters (a–f) were significantly different at a level of *p* < 0.05 (*n* = 3) by DMRT (Duncan’s multiple range test).

Considering the complexity of chemical constituents and their diverse mechanisms of action, evaluating the antioxidant potential of a sample based on a single method is inappropriate. Thus, three different assays including DPPH, ABTS, and FRAP were employed in the current study to assess and compare the antioxidant activities of different *P. sibiricum* extracts [32]. As shown in Figure 1, the EA fraction displayed the strongest scavenging effect on DPPH and ABTS free radicals assays with the IC_50_ values of 90.47 ± 3.17 µg/mL and 8.96 ± 0.21 µg/mL, respectively. This same fraction also showed the most prominent iron reducing ability among the five samples (PE, EA, n-Bu, WA, and CE), having a FRAP value of 2.59 ± 0.02 mM Fe^2+^/g. Moreover, in the three assays, the n-Bu fraction exhibited relatively higher antioxidant activities, followed by the PE fraction. However, the other extracts (WA, CE) showed modest antioxidant capacities. Based on the results from these three assays, the EA fraction exhibited the highest antioxidant activity as compared with the other four extracts. Hence, the EA fraction was selected for further research.

### 3.2. Total Phenolic and Flavonoid Content

Numerous studies have supported the hypothesis that polyphenols and flavonoids in plants are natural antioxidants that can exhibit antioxidant effects by strongly capturing free radicals like ROS. It was found that *P. sibiricum* possessed a good antioxidant activity, and its EA fraction had the highest scavenging effect on DPPH and ABTS free radicals. The TPC and TFC of five *P. sibiricum* extracts were assessed using Folin–Ciocalteu and the aluminum nitrate colorimetric methods. As shown in Table 1, TPC and TFC levels varied between *P. sibiricum* CE and its four fractions. The TPC of the EA fraction (144.736 ± 6.419 mg GAE/g) was the highest, followed by the n-Bu (19.143 ± 1.234 mg GAE/g) and PE (10.417 ± 0.899 mg GAE/g) fractions, and the CE has the weakest phenolic content of 0.543 ± 0.014 mg GAE/g.

Similarly, the EA fraction presented the most abundant flavonoids (119.204 ± 3.099 mg RT/g), followed by the n-Bu fraction (3.999 ± 0.381 mg RT/g), while the WA fraction (0.078 ± 0.015 mg RT/g) had the lowest. Consequently, it is speculated that the greatest antioxidant potentiality of the EA fraction could be attributed to its high levels of TPC and TFC.

### 3.3. Screening for SOD and XOD Ligands in P. sibiricum with UF-LC-MS

**Figure 2:**
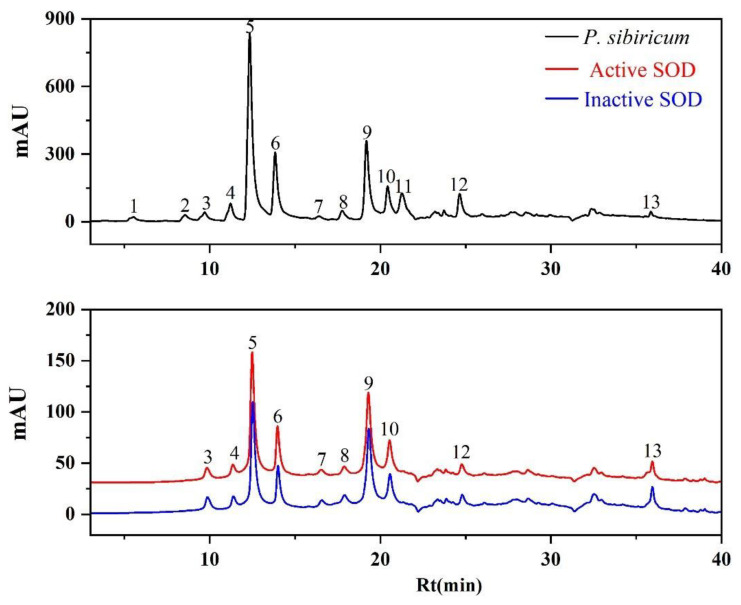
The UF-LC-UV chromatograms of the EA fraction from *P. sibiricum* with superoxide dismutase (SOD) at 280 nm. The black line represents the UPLC profiles of EA fraction, the red and blue line represent activate and inactivate SOD, respectively.

**Figure 3:**
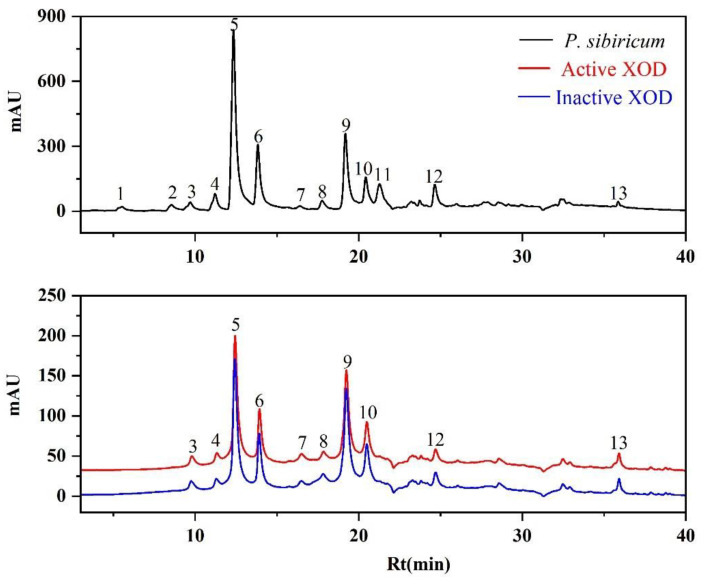
The UF-LC-UV chromatograms of the EA fraction from *P. sibiricum* with xanthine oxidase (XOD) at 280 nm. The black line represents UPLC profiles of EA fraction, the red and blue lines represent activate and inactivate XOD, respectively.

In previous studies, polysaccharides isolated from *P. sibiricum* were speculated as possible antioxidative and anti-hyperuricemic components on traditional animal experiments [28,29]. However, no substantial evidence for both its active components and their corresponding targets has been explored so far. To further explore the respective bioactive components in the EA fraction, the fast screening using bio-affinity ultrafiltration method with two targets (SOD and XOD) was applied to *P. sibiricum* [36,37]. The screened-out candidates might be regarded as potential SOD and XOD ligands contributing to the antioxidative and anti-hyperuricemic activities of the EA fraction. As shown in Figure 2 and Figure 3, the components released from the binding complex and collected in the ultrafiltrates were analyzed by UPLC, in which 10 and 4 peaks with various binding abilities to SOD and XOD were observed. Herein, in order to further evaluate the affinity between enzymes and ligands, the binding degree (BD) was counted by the following equation:

BD (%) = (Aa − Ab)/Aa × 100%(2)

where Aa and Ab represent the peak area of the active and inactive enzyme groups, respectively. If peak areas in active group were greater than that of inactive group, the components were deduced as potential inhibitors [41].

The BD of potential ligands in the EA fraction targeting SOD and XOD are summarized in Table 2. For SOD, peak 10 possessed the highest binding degree (25.21%), followed by peak 6 (24.28%), peak 5 (22.93%), peak 9 (18.81%), peak 3 (17.00%), peak 7 (16.90%), peak 4 (14.86%), peak 12 (14.83%), and other lower binding ability peaks. For XOD, peak 7 exhibited a strong binding to XOD with the highest BD value of 39.72%, followed by peak 8 (9.83%), peak 3 (8.86%), and peak 4 (7.21%). In fact, the peak areas of some components in the inactive group exceeded that of the active group, with BDs < 0, were not considered as XOD ligands. When the inactive enzyme with denatured conformation was co-incubated with the sample solution, some ultrafiltration membrane would be blocked. Therefore, some non-specific small molecules were retained on the ultrafiltration membrane throughout the elution process. After dissociation, relatively larger components were obtained, resulting in greater final detected peak areas and negative BDs [41,42]. Based on this, peaks 5, 6, and 10 were considered to as potential SOD ligands, and peak 7 were presumed to be XOD potential ligands for further study.

### 3.4. Identification of SOD and XOD Ligands in P. sibiricum with UPLC-Q-TOF-MS/MS

The SOD and XOD ligands that were screened out above were identified using the high-resolution analytical instrument UPLC-Q-TOF-MS/MS in the negative-ion mode. The structures of these compounds were assigned by comparison with the MS/MS fragments and retention times reported in previous studies [43,44,45], as well as the corresponding standards compounds. The retention time (Rt), quasi-molecular ion ([M-H]^−^ in negative ion mode), and characteristic fragments are listed in Table 2.

**Figure 4:**
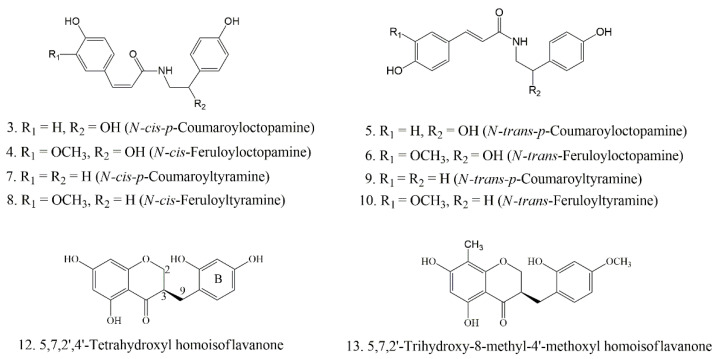
The potential ligands screened from the EA fraction of *P. sibiricum* by UF-LC-MS with superoxide dismutase (SOD) and xanthine oxidase (XOD).

Firstly, the peak 10 was identified as *N*-*trans*-feruloyltyramine and taken as an example to explain fragmentation details of the homologous structures. Peak 10 had a quasi-molecular ion [M-H]^−^ at *m/z* 312.1253 (C_18_H_18_NO_4_) and the fragment ions at *m/z* 148.0501 [M-H-C_9_H_10_NO_2_]^–^, 178.0482 [M-H-C_8_H_6_O_2_]^–^ exerted high intensity [43]. In addition, the fragment ions at *m/z* 190.0447 [M-H-C_7_H_6_O_2_]^–^, 135.0428 [M-H-C_10_H_11_NO_2_]^–^ corresponded to the deprotonated forms of *N*-[2-(4-hydroxyphenyl)ethyl] acrylamide (C_11_H_13_NO_2_), and dihidroxystyrene (C_8_H_8_O_2_), respectively [44]. The structure of peak 10 was further determined by the retention time and secondary fragments of the corresponding standard. Peak 8 was identified as *N*-*cis*-feruloyltyramine possessing the same quasi-molecular ion and fragment ion as peak 10, which were further distinguished by the retention time and reference data about cis/trans isomers [44,45]. Similarly, peaks 7 and 9 exhibited the same [M-H]^–^ ions at *m/z* 282.1144 (C_17_H_17_NO_3_), with 30 Da (a methoxyl group) less than those of peaks 8 and 10. In addition, peaks 7 and 9 produced characteristic fragment ions at *m/z* 119.0495 [M-H-C_9_H_9_NO_2_]^–^, with 16 Da (a hydroxyl group) less than those of peaks 8 and 10, which exhibited the same fracture mechanism as peaks 8 and 10. Thus, structures of peaks 7 and 9 were tentatively determined to be *N*-*cis*-*p*-coumaroyltyramine and *N*-*trans*-*p*-coumaroyltyramine. Peaks 3 and 5 exhibited [M-H]^–^ ions at *m/z* 298.1084 (C_17_H_17_NO_4_), with 16Da larger than those of peaks 7 and 9. The MS^2^ fragment ions at *m/z* 280.0968 [M-H-H_2_O]^–^, 145.0221 [M-H-H_2_O-C_8_H_7_O_2_]^–^, 119.0515 [M-H-H_2_O-C_9_H_7_NO_2_]^–^ was observed, which was deduced that an octopamine moiety in peaks 3 and 5 replaced the tyramine moiety in peaks 7 and 9 [44]. Hence, peaks 3 and 5 could be identified as *N*-*cis*-*p*-coumaroyloctopamine and *N*-*trans*-*p*-coumaroyloctopamine [45], which were already confirmed by the standard compound. Based on a similar principle, peaks 4 and 6, with the same [M-H]^–^ ion at *m/z* 328.1197, were detected as *N*-*cis*-feruloyloctopamine and *N*-*trans*-feruloyloctopamine, respectively, and the latter was confirmed by the corresponding standard. The C_2-3_ bond saturation status and the type of substituted groups on the B-ring related to the fragmentation behaviors of homoisoflavonoids [46,47]. When the C_2-3_ bond was saturated and the B-ring was replaced with a hydroxyl group, the predominant fragmentation was [M-H-CH_2_-B-ring+H]^–^, which was subsequently followed by the neutral loss of a carbonyl group to obtain [M-H-CH_2_-B-ring+H-CO]^–^. Based on this principle, peak 12 ([M-H]^−^, *m/z* 301.0695) produced a characteristic MS^2^ fragment ion at *m/z* 179.0334 ([M-H-CH_2_-B-ring(C_7_H_8_O)]^−^, and a fragment ion at *m/z* 125.0243, which was unequivocally identified as 5,7,2’,4’-tetrahydroxyl homoisoflavanone [48], while peak 13 was tentatively determined to be 5,7,2’-trihydroxy-8-methyl-4’-methoxyl homoisoflavanone [44]. Their structures are shown in Figure 4. The secondary mass spectrometry fragments of the three standard compounds with high binding degree to SOD enzyme as potential bioactive ligands and the retention time are shown in Figures S2–S5, which are completely consistent with our identification results.

### 3.5. Molecular Docking

Molecular docking has been commonly performed to evaluate the interaction between the enzyme and the potential ligand and reveal the possible interaction modes by determining their docking energy, site of action, and the contributive key residues of the receptors [49,50].

**Figure 5:**
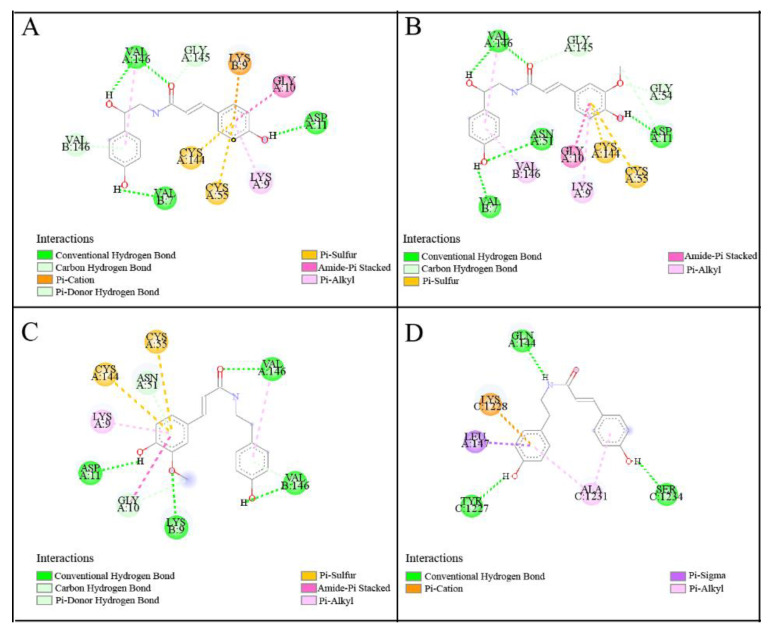
Docking models of screened ligands with SOD and XOD: (**A**), SOD-*N*-*trans*-*p*-coumaroyloctopamine; (**B**), SOD-*N*-*trans*-feruloyloctopamine; (**C**), SOD-*N*-*trans*-feruloyltyramine; (**D**), XOD-*N*-*cis*-*p*-coumaroyltyramine, respectively.

In this work, according to the BD values of the potential ligands, the three compounds (peak 5, 6, 10) were docked with SOD and one compound (peak 7) to XOD. The binding energy (BE), inhibition constant (Ki), and hydrogen bonds are summarized in Table 3, and their best docking conformations within the binding sites are illustrated in Figure 5. Dithiocarbamate (DTC) and allopurinol (ALL) were set as positive controls towards SOD and XOD, correspondingly. For SOD, the three potential ligands, *N*-*trans*-*p*-coumaroyloctopamine, *N*-*trans*-feruloyloctopamine, and *N*-*trans*-feruloyltyramine, could smoothly enter the active pocket with low BE values. Thereinto, *N*-*trans*-feruloyltyramine (peak 10) exhibited the strongest affinity to SOD with the lowest binding energy (BE) of −6.83 kcal/mol and the inhibition constant (Ki) of 9.83 μM, followed by peak 6 (BE, −6.58 kcal/mol; Ki, 14.97 μM) and peak 5 (BE, −6.54 kcal/mol; Ki, 16.13 μM). Meanwhile, the BEs and Kis of peak 5 (*N*-*trans*-*p*-coumaroyloctopamine), peak 6 (*N*-*trans*-feruloyloctopamine), and peak 10 (*N*-*trans*-feruloyltyramine) were lower than the positive control DTC (BE, −3.84 kcal/ mol; Ki, 1.52 mM), which showed that all the screened compounds have strong interactions with SOD. Particularly, the molecular docking results were in full compliance with the ultrafiltration screening results, with a relatively large BD value displaying a good affinity to SOD. Figure 5A revealed that *N*-*trans*-*p*-coumaroyloctopamine could form three hydrogen bonds with the active amino acid residue Val146, Val7 and Asp11. From Figure 5B, four hydrogen bonds were formed between *N*-*trans*-feruloyloctopamine and the active pocket of SOD (Val146, Val7, Asp11, Asn51). As Figure 5C showed that *N*-*trans*-feruloyltyramine could interact with SOD by forming four hydrogen bonds with the residues of Val146, Lys9, and Asp11. With regard to XOD, *N*-*cis*-*p*-coumaroyltyramine (peak 7) displayed a higher affinity to XOD with a lower BE of −6.51 kcal/mol, and the inhibition constant (Ki) of 16.83 μM, which was better than the positive drug ALL (BE, −5.08 kcal/mol; Ki, 189.72 μM). Three hydrogen bonds were formed between the amino acid residues of XOD (Gln144, Tyr1227, Ser1234) and *N*-*cis*-*p*-coumaroyltyramine in Figure 5D. Hydrogen bonds were the main contributor to stabilize the complex formed with these ligands-targets, moreover, there are some other forces including carbon hydrogen bond, pi–sigma, amide–pi stacked, pi–alkyl, and so on, which also contributed to the interactions between the two enzymes and those potential active ligands.

In summary, the molecular docking study confirmed that *N*-*trans*-*p*-coumaroyloctopamine, *N*-*trans*-feruloyloctopamine, *N*-*trans*-feruloyltyramine, and *N*-*cis*-*p*-coumaroyltyramine interact well with SOD and XOD, respectively. Meanwhile, their BEs and Kis values were found better than the positive controls, which theoretically indicates that these compounds had a high inhibition effect on SOD and XOD. Hence, *N*-*trans*-*p*-coumaroyloctopamine, *N*-*trans*-feruloyloctopamine, and *N*-*trans*-feruloyltyramine were identified as potential ligands for SOD, and *N*-*cis*-*p*-coumaroyltyramine was determined as the potential ligand for XOD, whose activity was studied further.

### 3.6. Antioxidant Capacity of Potential Ligands by UF-LC-MS

**Figure 6:**
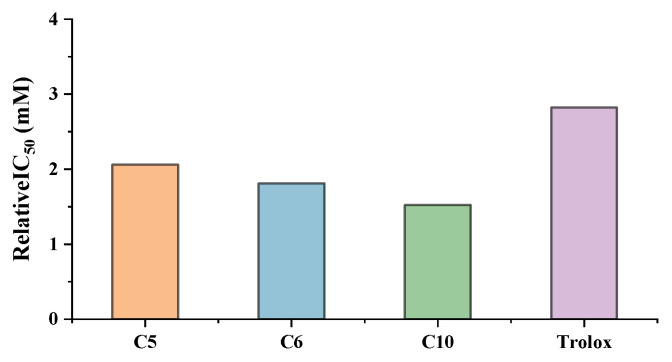
The relative IC_50_ of potential ligands with SOD. C5: *N*-*trans*-*p*-coumaroyloctopamine; C6: *N*-*trans*-feruloyloctopamine; C10: *N*-*trans*-feruloyltyramine.

The relative IC_50_ of potential ligands with SOD was determined using UF-LC-MS, and the results are shown in Figure 6. The UF-LC-MS chromatogram is presented in Figure S1. As shown in Figure 6, compound 10 (*N*-*trans*-feruloyltyramine, relative IC_50_ = 1.52 mM) exhibits the highest SOD activity, followed by compound 6 (*N*-*trans*-feruloyloctopamine, relative IC_50_ = 1.81 mM) and compound 5 (*N*-*trans*-*p*-coumaroyloctopamine, relative IC_50_ = 2.06 mM). In addition, all three compounds showed better antioxidant activity than the positive control (Trolox). Their inhibition trend was consistent with the molecular docking and ultrafiltration screening results, with relatively large BD values displaying stronger inhibition to SOD.

## 4. Conclusions

In the present study, the potential bioactive ligands with noteworthy antioxidative and anti-hyperuricemic activities together with their respective mechanisms of action were revealed for the first time from *P. sibiricum.* On the one hand, the antioxidant capacity of *P. sibiricum* was firstly evaluated with the DPPH, ABTS and FRAP assays, and the EA fraction exhibited the highest DPPH, ABTS radical scavenging and ferric reducing capacities with the IC_50_ values of 90.47 ± 3.17 µg/mL, 8.96 ± 0.21 µg/mL and a FRAP value of 2.59 ± 0.02 mMFe^2+^/g. On the other hand, the EA fraction showed the highest total phenolic and flavonoid contents of 144.74 ± 6.42 mg GAE/g dw and 119.20 ± 3.10 mg RT/g dw among other extracts from *P. sibiricum*. Then, the bio-affinity ultrafiltration combining SOD and XOD with LC-MS/MS was used to screen out its potential bioactive antioxidative and anti-hyperuricemic compounds from *P. sibiricum*. As a result, 10 and 4 peaks with various binding abilities to SOD and XOD were explored based on their BD values. Peak 5 (*N*-*trans*-*p*-coumaroyloctopamine), peak 6 (*N*-*trans*-feruloyloctopamine), and peak 10 (*N*-*trans*-feruloyltyramine) were presumed to be potential ligands to SOD, and peak 7 (*N*-*cis*-p-coumaroyltyramine) was identified as the potential ligand to XOD. Additionally, molecular docking showed that these four compounds effectively interacted with SOD and XOD by hydrogen bonds and other varied interaction forces, and even were better than the corresponding positive controls (DTC and ALL). In summary, this study not only provides new evidence to support the antioxidative and anti-hyperuricemic pharmacological activities of *P. sibiricum,* but also offers the respective potential bioactive ligands from *P. sibiricum* for further study and application. More importantly, to the best of our knowledge, this is the first study to rapidly screen and identify the antioxidative and anti-hyperuricemic bioactive ligands from *P. sibiricum* targeting SOD and XOD through the UF-LC-MS method. These bioactive compounds have the potential to be applied in the development of *P. sibiricum* as a functional food or natural medicine against oxidization-related and hyperuricemia-related diseases in the near future.

## Figures and Tables

**Table 1:** Total phenolic and total flavonoid contents of *P. sibiricum*.

Extracts	TPC (mg GAE/g dw)	TFC (mg RT/g dw)
PE	10.417 ± 0.899 ^c^	2.859 ± 0.106 ^b^
EA	144.736 ± 6.419 ^a^	119.204 ± 3.099 ^a^
n-Bu	19.143 ± 1.234 ^b^	3.999 ± 0.381 ^b^
WA	0.790 ± 0.015 ^d^	0.078 ± 0.015 ^c^
CE	0.543 ± 0.014 ^d^	0.188 ± 0.018 ^c^

Data are expressed as means ± SD. Means labeled by different letters (a–d) were significantly different at a level of *p* < 0.05 (*n* = 3) by DMRT (Duncan’s multiple range test). TPC, total phenolic content; TFC, total flavonoid content; GAE/g dw, gallic acid equivalent per gram of dry weight; RE/g dw, rutin equivalent per gram of dry weight. Petroleum ether (PE), ethyl acetate (EA), n-butanol (n-Bu), aqueous (WA) and crude extracts (CE) of *P. sibiricum*.

**Table 2:** The identification, binding degree (BD) and the UF-LC-MS/MS data of potential SOD and XOD ligands screened out from *P. sibiricum*.

NO.	Rt/min	[M-H]^−^ (*m/z*)	MS/MS Spectrum	Identification	BD (%)	
					SOD	XOD
3	9.71	298.1008	280.0918, 145.0272, 119.0483	*N*-*cis*-*p*-Coumaroyloctopamine	17.00	8.86
4	11.22	328.1114	310.1057, 161.0235, 133.0521	*N*-*cis*-Feruloyloctopamine	14.86	7.21
5	12.34	298.1032	280.0968, 145.0281, 119.0515	*N*-*trans*-*p*-Coumaroyloctopamine	22.93	−3.29
6	13.83	328.1209	310.1028, 161.0230, 133.0528	*N*-*trans*-Feruloyloctopamine	24.28	−1.01
7	16.44	282.1144	162.0504, 145.0221, 119.0468	*N*-*cis*-*p*-Coumaroyltyramine	16.90	39.72
8	17.78	312.1246	190.0471, 178.0462, 148.0502, 135.0434	*N*-*cis*-Feruloyltyramine	6.95	9.53
9	19.20	282.1146	162.0539, 119.0495	*N*-*trans*-*p*-Coumaroyltyramine	18.81	−6.76
10	20.43	312.1253	190.0447, 178.0482, 148.0501, 135.0428	*N*-*trans*-Feruloyltyramine	25.21	−0.96
12	24.66	301.0695	179.0334, 125.0243	5,7,2’,4’-Tetrahydroxyl homoisoflavanone	14.83	−1.48
13	35.82	329.1057	193.0499, 139.0411	5,7,2’-Trihydroxy-8-methyl-4’- methoxyl homoisoflavanone	7.19	0.99

NO., the number of chromatographic peaks; Rt, retention time; SOD, superoxide dismutase; XOD, xanthine oxidase.

**Table 3:** The molecular docking results of the potential ligands in *P. sibiricum* with SOD and XOD.

Peak	SOD (PDB 1CBJ)			XOD (PDB 1FIQ)		
	BE (kcal/mol)	Ki	Hydrogen Bonds	BE (kcal/mol)	Ki	Hydrogen Bonds
5	−6.54	16.13 μM	Val146, Val7, Asp11	ND	ND	ND
6	−6.58	14.97 μM	Val146, Val7, Asp11, Asn51	ND	ND	ND
7	ND	ND	ND	−6.51	16.83 μM	Gln144, Tyr1227, Ser1234
10	−6.83	9.83 μM	Val146, Lys9, Asp11	ND	ND	ND
DTC ^a^	−3.84	1.52 mM	Gly49, Asn51, Val5	ND	ND	ND
ALL ^b^	ND	ND	ND	−5.08	189.72 μM	Lys1228, Glu1210

PDB, Protein Data Bank; 1CBJ, the crystal structure accession number of SOD; 1FIQ, the crystal structure accession number of XOD; BE, binding energy; Ki, inhibition constant; a, Dithiocarbamate (DTC), positive control of SOD; b, Allopurinol (ALL), positive control of XOD; ND, not detected; Val, Valine; Asp, aspartic acid; Asn, asparaginate; Lys, Lysine; Gln, glutamine; Tyr, tyrosine; Ser, serine; His, histidine; Phe, phenylalanine; Ala, alanine.

## Data Availability

All data in this study are included in this article and Supplementary Materials.

## Supplementary Materials

The following supporting information can be downloaded at: https://www.mdpi.com/article/10.3390/antiox11091651/s1, Figure S1: The UF-LC-UV chromatograms of the potential ligands of superoxide dismutase (SOD) at 280 nm; Figure S2: The mass spectrometry fragments of *N*-*trans*-*p*-coumaroyloctopamine in standard substance and *P. sibiricum*; Figure S3: The mass spectrometry fragments of *N*-*trans*-feruloyloctopamine in standard substance and *P.sibiricum*; Figure S4: The mass spectrometry fragments of *N*-*trans*-feruloyltyramine in standard substance and *P. sibiricum*; Figure S5: The UPLC of the potential ligands’ standards to SOD. A: *P. sibiricum*; B: *N*-*trans*-*p*-coumaroyloctopamine; C: *N*-*trans*-feruloyloctopamine; D: *N*-*trans*-feruloyltyramine; E: *N*-*trans*-*p*-coumaroyloctopamine + *N*-*trans*-feruloyloctopamine + *N*-*trans*-feruloyltyramine; Table S1: The relative binding degree (BD) and the relative IC_50_ data of potential SOD ligands in *P. sibiricum*.
