# Supplementary material for: Potential Antioxidative and Anti-Hyperuricemic Components Targeting Superoxide Dismutase and Xanthine Oxidase Explored from Polygonatum Sibiricum Red
Source: Antioxidants (Basel). 2022 Aug 25;11(9):1651. doi: 10.3390/antiox11091651 (PMC9495925; doi:10.3390/antiox11091651)
Supplement: Supplementary file 1 [file antioxidants-11-01651-s001.zip › antioxidants-1868529-supplementary.pdf]

antioxidants

Supplementary materials

Potential Antioxidative and Anti-Hyperuricemic Components Targeting Superoxide Dismutase and Xanthine Oxidase Explored from *Polygonatum Sibiricum* Red.

Jing Li [1,2,†], Zhi Wang [3,†], Minxia Fan [1,4,5], Guangwan Hu [1,2,4,5,*], and Mingquan Guo [1,2,4,5,*]

[1] Key Laboratory of Plant Germplasm Enhancement and Specialty Agriculture, Wuhan Botanical Garden, Chinese Academy of Sciences, Wuhan 430074, China
[2] College of Life Sciences, University of Chinese Academy of Sciences, Beijing 100049, China
[3] College of Pharmacy, Hunan University of Chinese Medicine, Changsha 410208, China
[4] Sino-Africa Joint Research Center, Chinese Academy of Sciences, Wuhan 430074, China
[5] Innovation Academy for Drug Discovery and Development, Chinese Academy of Sciences, Shanghai 201203, China
* Correspondence: guangwanhu@wbgcas.cn (G.H.); guomq@wbgcas.cn (M.G.); Tel.: +86-027-87700850
† These authors contributed equally to this work.

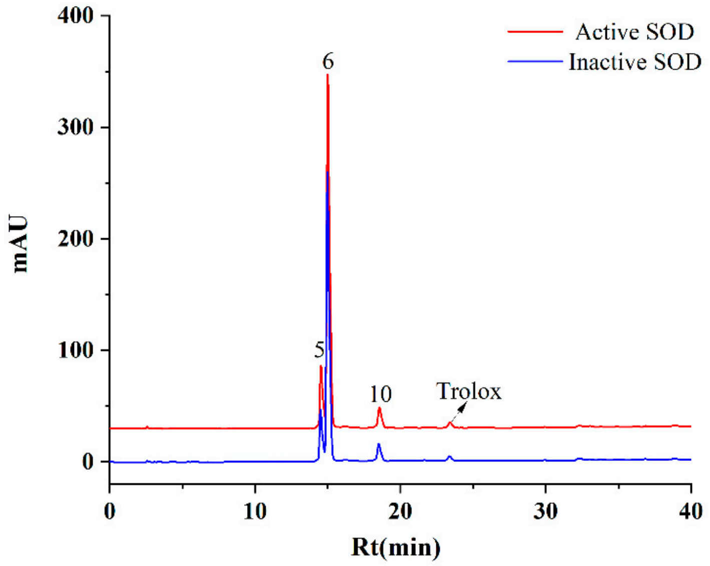

Figure S1. The UF-LC-UV chromatograms of the potential ligands of superoxide dismutase (SOD) at 280 nm. The red and blue line represent activated and inactivated SOD, respectively. (5, *N-trans-p*-coumaroyloctopamine; 6, *N-trans*-feruloyloctopamine; 10, *N-trans*-feruloyltyramine).

Table S1. The relative binding degree (BD) and the relative IC_{50} data of potential SOD ligands in *P. sibiricum*.

NO.	Compound	BD (%)	Relative IC_{50} (mM)
5	*N-trans-p*-coumaroyloctopamine	19.10	2.06
6	*N-trans*-feruloyloctopamine	21.70	1.81
10	*N-trans*-feruloyltyramine	25.80	1.52
-	Trolox	13.98	2.82

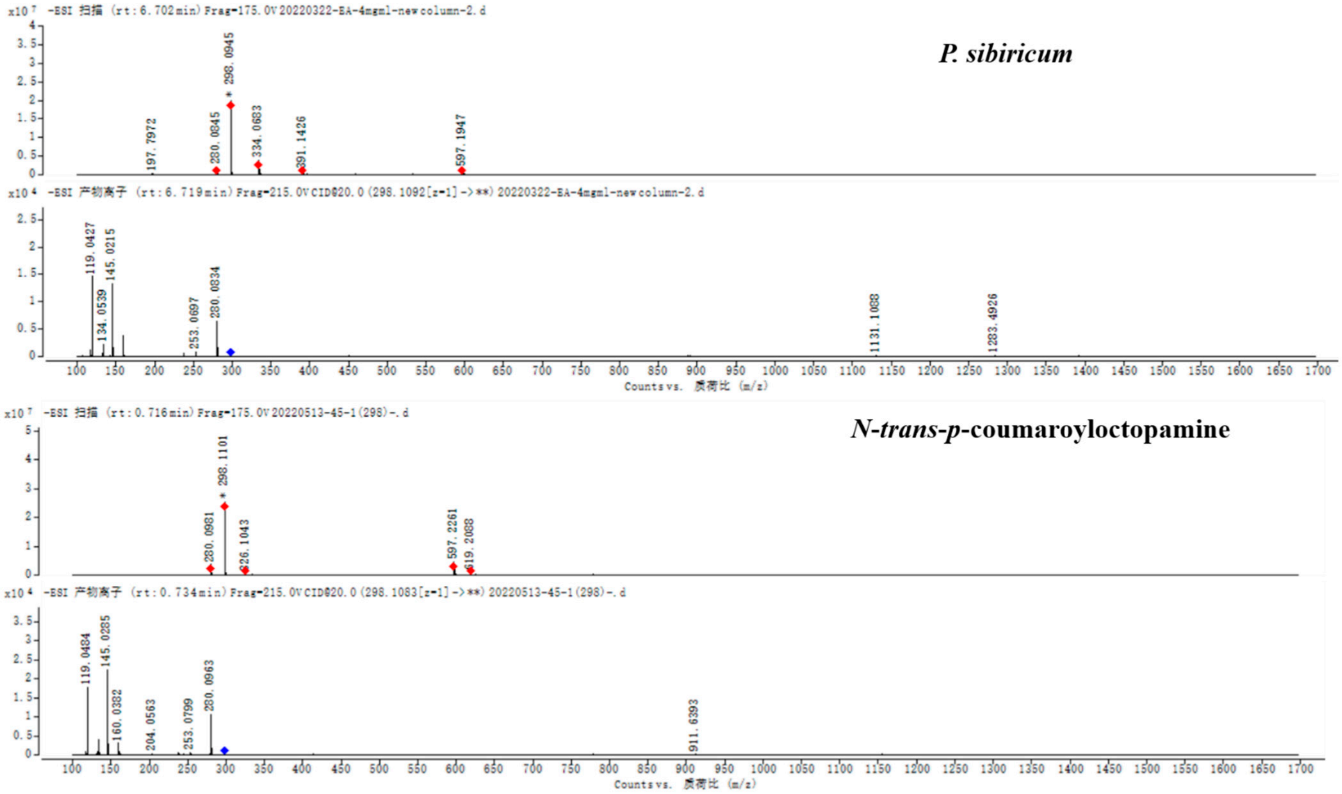

Figure S2. The mass spectrometry fragments of *N-trans-p*-coumaroyloctopamine in standard substance and *P. sibiricum*.

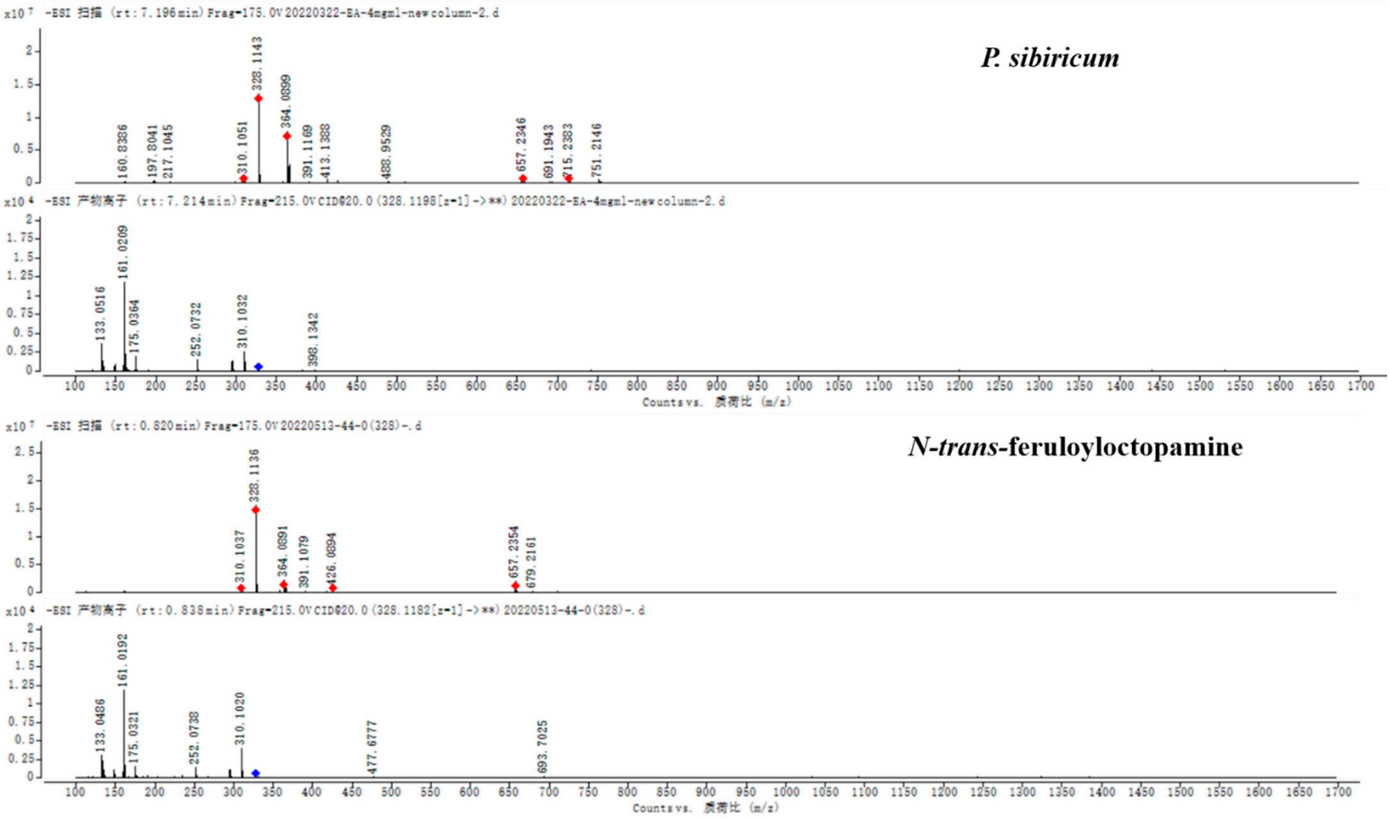

Figure S3. The mass spectrometry fragments of *N-trans*-feruloyloctopamine in standard substance and *P. sibiricum*.

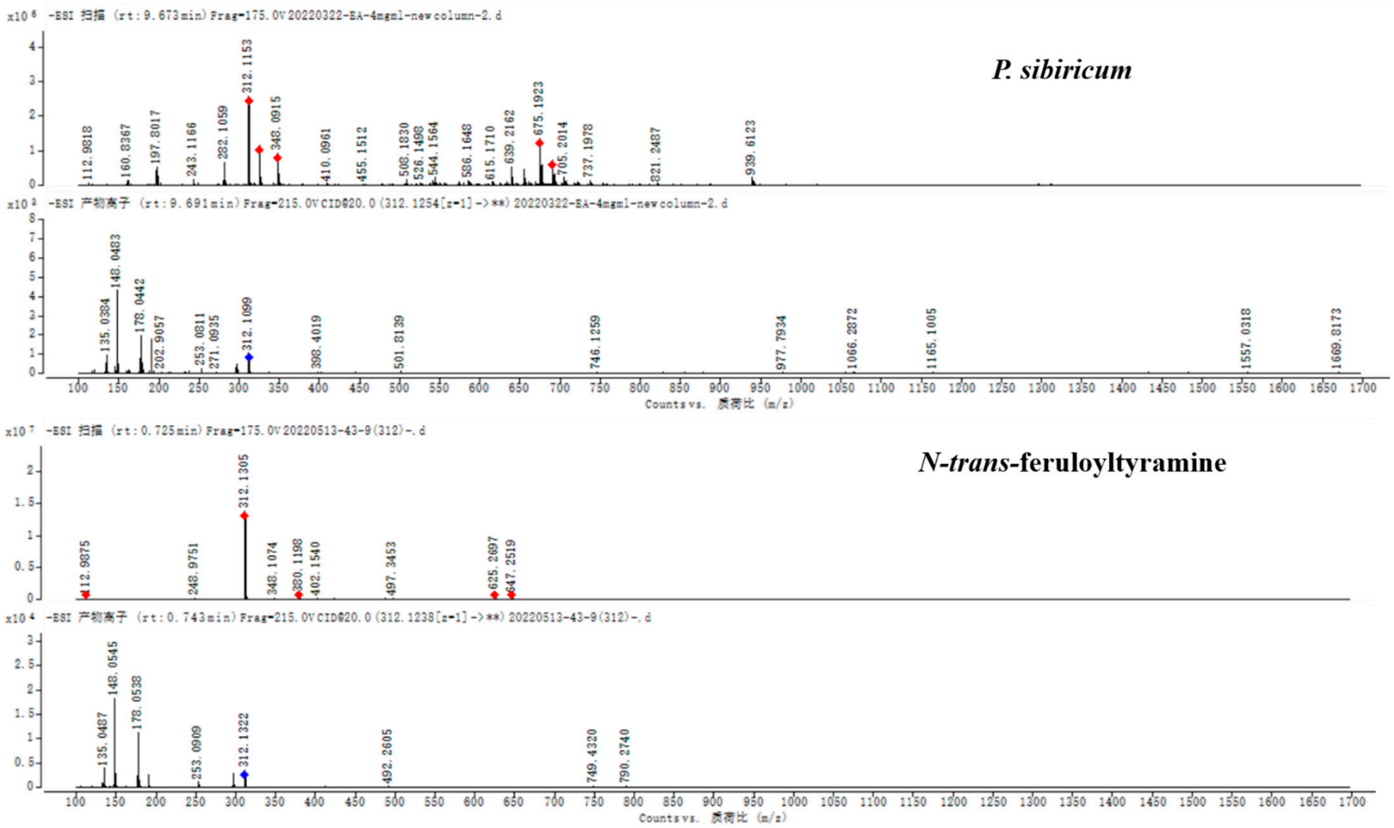

Figure S4. The mass spectrometry fragments of *N*-*trans*-feruloyltyramine in standard substance and *P. sibiricum*.

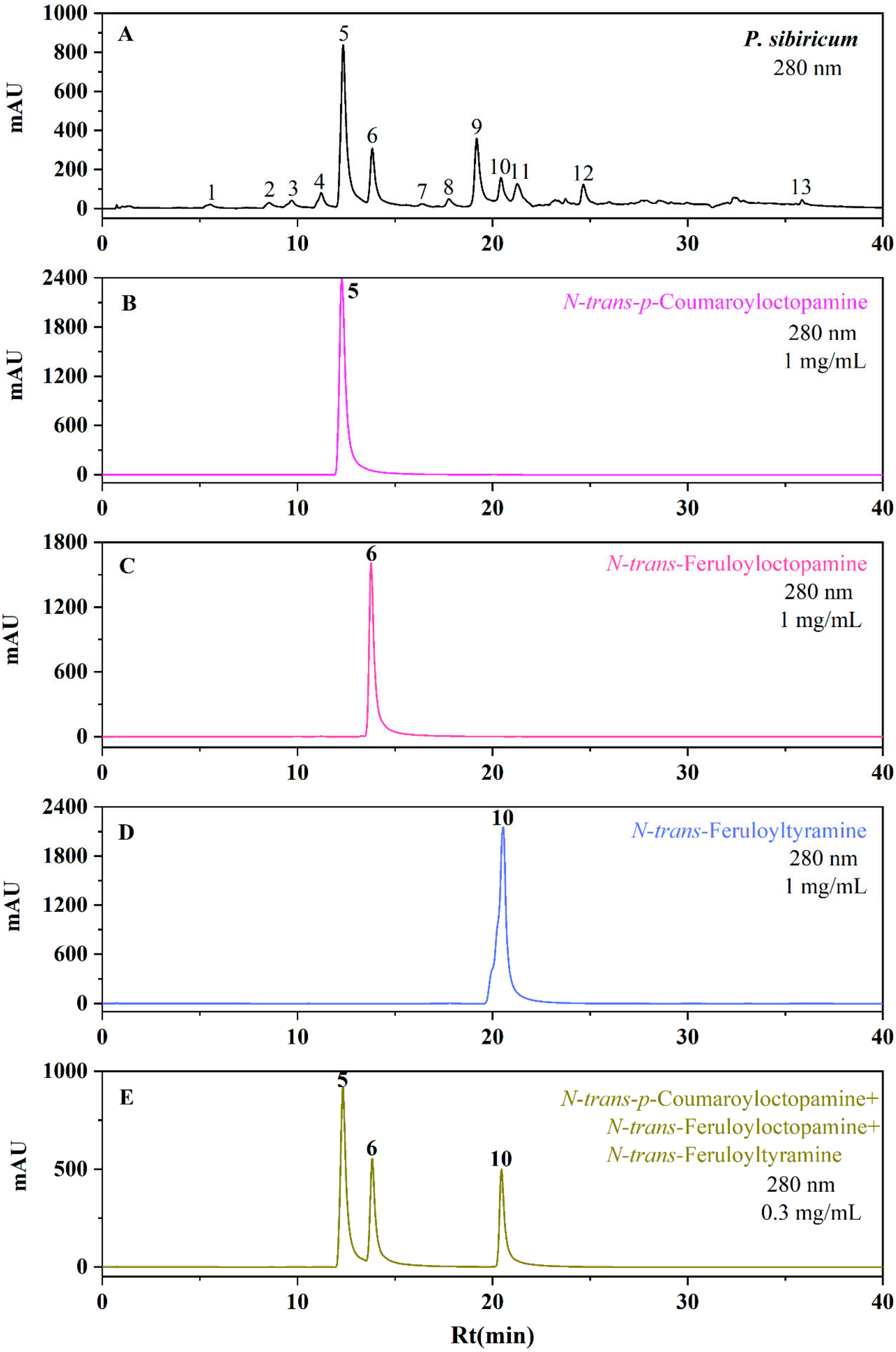

Figure S5. The UPLC of the potential ligands' standards to SOD. A: *P. sibiricum*; B: *N*-*trans*-*p*-coumaroyloctopamine; C: *N*-*trans*-feruloyloctopamine; D: *N*-*trans*-feruloyltyramine; E: *N*-*trans*-*p*-coumaroyloctopamine + *N*-*trans*-feruloyloctopamine + *N*-*trans*-feruloyltyramine.
